# Supplementary material for: The Synchronized Progression from Mitosis to Meiosis in Female Primordial Germ Cells between Layers and Broilers
Source: Genes (Basel). 2023 Mar 23;14(4):781. doi: 10.3390/genes14040781 (PMC10137798; doi:10.3390/genes14040781)
Supplement: Supplementary file 1 [file genes-14-00781-s001.zip › genes-2250108-supplementary.pdf]

The synchronized progression from mitosis to meiosis in female primordial germ cells between layers and broilers

Yuxiao Ma , Wenhui Wu , Yun Zhang , Xuzhao Wang , Jiahui Wei , Xiaotong Guo , Man Xue and Guiyu Zhu*

College of Animal Science and Technology, Shandong Provincial Key Laboratory of Animal Biotechnology and Disease Control and Prevention, Shandong Agricultural University, Taian, China.
*Correspondence: zhuguiyu@sdau.edu.cn

Table S1. Quantitative real-time PCR primer sequences used in this study

Gene	Sense primer	Anti-sense primer
MCM2	AGGATCAGCGACATGTGCAA	ACTTGTTACCACTCCGCTGG
CDKN2A	TGACCTCTCGGATAAGGTGC	CTCCTTCTCAGAACCCGGC
IL4I1	AATCACGGGAGGTTTCGACC	AGAACTGGATGTGCCTCGTG
CSF3	AGTTCACCCGCAAGATCAGG	TCCACCAACGTCGTGTGATT
PROK2	ACGTAGGAGAGGAGTGCCAT	CACCTGAACTTGCTGGGTGA
IGF2	TCAGTAGACCAGTGGGACGA	CGGACTTGGCACAGTAGGTT
E2F4	ACGTGCAGAACAACTGGCTA	GATGGGAACCTCTAGGCGTG
E2F6	TCTGAGCGCGACACCAA	TCCAAGAGCTGTTGCTACGTC
CDKN1B	CCGACTTCTACTTCAGGCAG	GCAATTCCCGTTTACATCCAG
BNIP3	AATGGGAATGGCAATGGAAAC	TGTGAATGGAGATAGAAGCTGG
STRA8	CCAGTGGTTCCTTGGTCTCC	ATCATCGAAGGTCTCCGTGC
RALDH2	ATCAAGGAGGCTGGCTTTCC	TCCTTCCAGCTGCTTCTTGG
RAD54L	ATGGCCAGAAGAAGACCTGC	ACACAGCGACGACACTTGAT
TET2	GCGGAGATACGTTCAGAGGG	GCTTCACTGCAGAAGGGTCT
GAPDH	GAGGGTAGTGAAGGCTGCTG	CACAACACGGTTGCTGTATC

Table S2. Statistics of sequencing data for PGCs

Sample name	Clean reads	Clean GC percent(%)	Q30(%)	Total mapped(%)
H10-1	44,902,888	50.19	91.15	95.33
H10-2	42,241,266	50.34	91.63	95.74
H14-1	38,920,932	49.83	91.06	94.72
H14-2	40,039,976	49.62	91.51	95.17
Y10-1	44,617,333	49.85	95.30	96.65
Y10-2	44,177,769	49.70	94.30	96.64
Y14-1	47,077,657	49.61	93.86	96.28
Y14-2	46,257,825	49.63	93.79	96.45

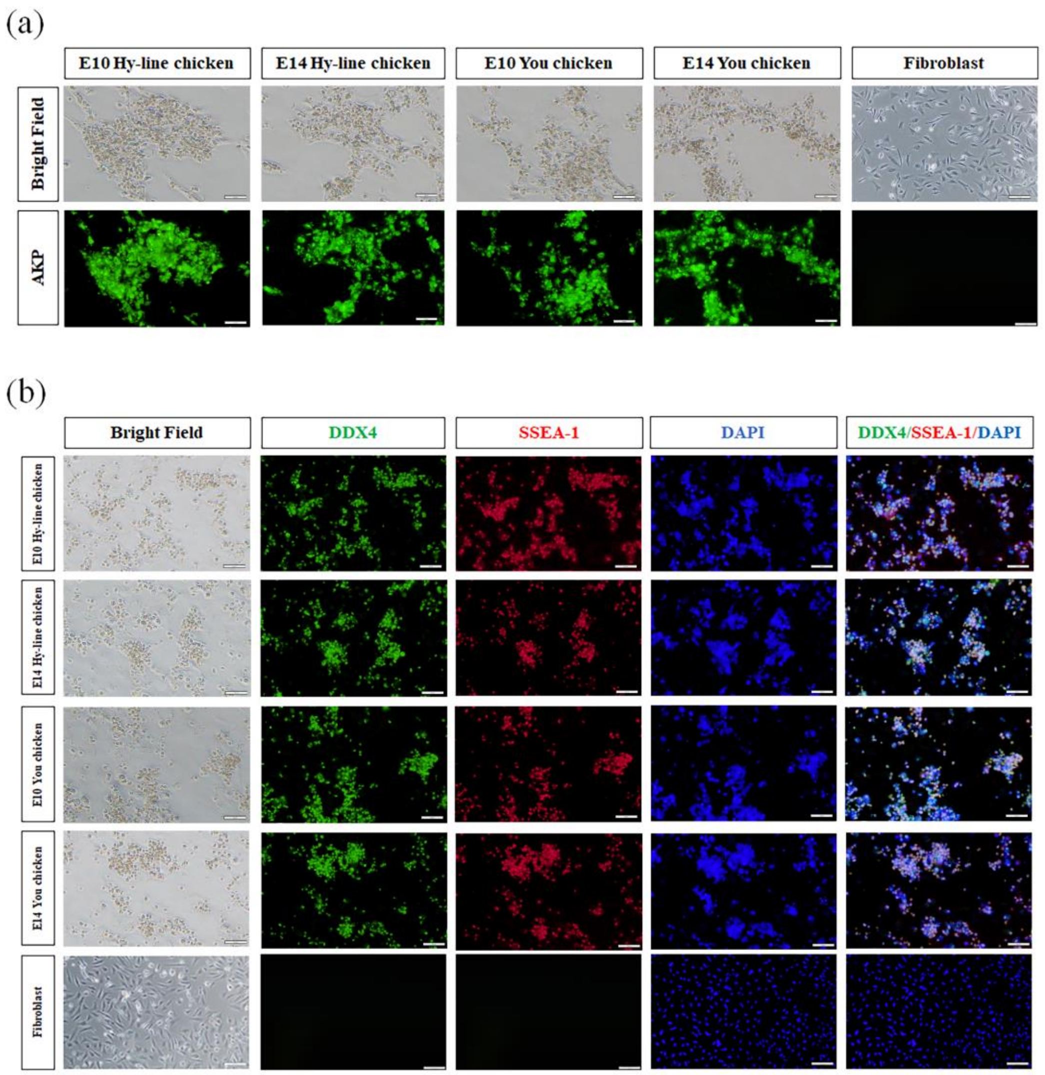

Figure S1. Cultivation and characterization of PGCs .(a) Representative pictures of AKP staining in PGCs and chicken embryonic fibroblast cells (CEFs). PGCs are green after staining with the alkaline phosphatase reagent. Scale bar: 50 μm. (b) The germ cell-specific marker DDX4 and embryonic stem cell marker SSEA-1 were positive on the isolated chicken PGCs assayed by immunofluorescence staining. The fibroblast cells were used as negative controls. Scale bars: 50 μm.

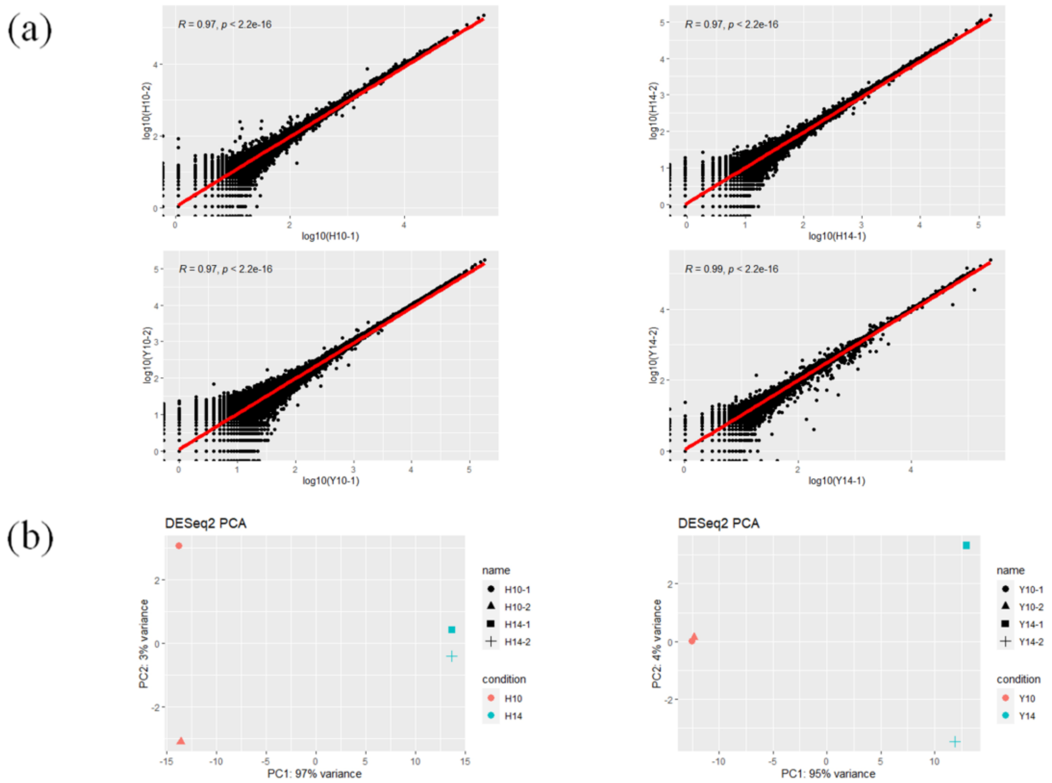

Figure S2. Comparison of gene expression for PGCs at different developmental stages. (a) Pearson correlation analysis between the repeated samples within the same group of PGCs. The x- and y-axes represent mean gene expression calculated by log 10 (FPKM+1). (b) PCA for PGCs from two developmental stages in both Hy-Line chicken (left) and You chicken (right). FPKM: fragments Per Kilobase of exon model per Million mapped fragments.
